# Polylactic Acid—Lemongrass Essential Oil Nanocapsules with Antimicrobial Properties

**DOI:** 10.3390/ph9030042

**Published:** 2016-07-07

**Authors:** Ioannis L. Liakos, Alexandru Mihai Grumezescu, Alina Maria Holban, Iordache Florin, Francesca D’Autilia, Riccardo Carzino, Paolo Bianchini, Athanassia Athanassiou

**Affiliations:** 1 Smart Materials Group, Nanophysics Department, Istituto Italiano di Tecnologia (IIT), Via Morego 30, Genoa 16163, Italy; Riccardo.Carzino@iit.it (R.C.); Athanassia.Athanassiou@iit.it (A.A.); 2 Department of Science and Engineering of Oxide Materials and Nanomaterials, Faculty of Applied Chemistry and Materials Science, University Politehnica of Bucharest, Polizu Street No. 1-7, Bucharest 011061, Romania; grumezescu@yahoo.com (A.M.G.); alina_m_h@yahoo.com (A.M.H.); 3 Department of Microbiology and Immunology, Faculty of Biology, University of Bucharest, Aleea Portocalelor, No. 1-3, Bucharest 060101, Romania; 4 Nicolae Simionescu Institute of Cellular Biology and Pathology, Bucharest, Romania; floriniordache84@yahoo.com; 5 Nanobiophotonics, Nanophysics Department, Istituto Italiano di Tecnologia (IIT), Via Morego 30, Genoa 16163, Italy; Francesca.Dautilia@iit.it (F.D.); Paolo.Bianchini@iit.it (P.B.)

**Keywords:** polylactic acid, lemongrass essential oil, antimicrobial nanocapsules, biofilms, infectious diseases

## Abstract

Polylactic acid was combined with lemongrass essential oil (EO) to produce functional nanocapsules (NCs). The obtained polylactic acid nanoparticles showed antimicrobial activity both with and without the presence of lemongrass oil; however, the presence of EO improved the activity of the NCs. The presence of lemongrass assisted the formation of well-separated NCs and also provided enhanced antimicrobial properties, since lemongrass is known for its antimicrobial character. Fluorescence microscopy was used to optically observe the nanoparticles and NCs and revealed the attachment of lemongrass oil with the polylactic acid NCs. Dynamic light scattering was used to determine their size. UV absorption was used to determine the exact amount of lemongrass oil found in the polylactic acid—lemongrass oil NCs, which was important for understanding the minimum inhibitory concentration for the antimicrobial experiments. A series of clinically important microbial species were used in the study and the obtained NCs proved to have very good antimicrobial properties against all tested strains. Such NCs can be used for the design of ecological strategies, based on natural alternatives, which may be efficient against severe infections, including those that involve resistant pathogens and biofilms or those with difficult to reach localization.

## 1. Introduction

Treatment of severe diseases such as those including difficult to reach pathogens, intracellular bacteria and increased microbial resistance require the development of antimicrobial approaches on the nanometer scale [1,2,3,4] to specifically target the infection. Nanoparticles (NPs) and nanocapsules (NCs), for example, can enter the alveoli of lungs with diameter few micrometers [5] and treat lung infections and other difficult to reach infections. Moreover, the NPs and NCs have high surface area to volume ratio [6] and thus can be more efficient in stopping microbial growth with only small amounts [7], can stabilize the pharmacological active compound by avoiding its unspecific spread within the body, ensure a targeted activity and a controlled release of the antimicrobial drug, and avoid the toxicity problems related with the use of high amounts of antibiotics that are usually necessary to solve severe infections, which is very important [8].

The polymers that are used as matrices in the preparation of NCs have different lifespans inside biological tissues [9,10]. For example, the polylactic acid (PLA) used in this work has a lifespan of about 9–12 months in contact with biological tissue, and, thus, can be important for the treatment of chronic diseases, such as respiratory, ophthalmic or ear infections and also those that involve resistant microbial infections or need a systemic treatment with antibiotics.

PLA is a biodegradable and biocompatible linear aliphatic polyester obtained from lactic acid monomers [11] originated usually from natural sources such as starch, sugarcane, etc. PLA has been used for biomedical applications such as wound sutures [12], tissue regeneration [13], orthopedic implants [13,14], drug encapsulation into PLA nanoparticles (NPs) [11,15], silver NP encapsulation into PLA nanocomposites with antibacterial properties [16], and others. More analytically, anthraquinone, an antibacterial agent, was encapsulated into chitosan/PLA NCs and found to have antimicrobial properties against *Escherichia coli*, *Pseudomonas aeruginosa*, *Klebsiella pneumoniae* and *Proteus vulgaris* [15]. Silver NPs were also encapsulated into hydrolyzed collagen/PLA NCs and found to retain the antimicrobial properties against *Candida albicans*. PLA with other polymers such as polyethylene glycol in the presence of poloxamer 188 surfactant has also been used to encapsulate halofatrine in *Plasmodium bergei* infected mice and found to treat such infection [17]. Poly (dl-lactide-*co*-glycolide) (PLGA) NPs with encapsulated and trans-cinnamaldehyde in the presence of polyvinyl alcohol also showed antimicrobial activity against *Salmonella* spp. and *Listeria* spp. [18]. Anticancer drugs, such as 5-fluorouracil and 6-thioguanine, were used to functionalize polyethylene terephthalate-polylactic acid copolymer NCs in the presence and absence of gold and iron oxide, where the presence of metallic NPs enhanced the anticancer activity of the NCs with limited harmful effects for healthy tissues [19]. *Picrorhizakurroa* root extract was encapsulated into PLA NPs with the use of pluronic-F-68 surfactant using the nanoprecipitation method as food supplements with nutraceutical value [20].

In the current work, we have used the nanoprecipitation method to obtain PLA/Lemongrass (LG) essential oil NCs without the use of additional polymers or surfactants. LG essential oils originate from steam distillation of lemongrass plant [21], and it is known for its antimicrobial properties [22] even when encapsulated into polymers [7,23,24,25]. LG essential oil consists of many molecules, such as hydrocarbons, aldehydes, alcohols, ketones and others [22,26]. Thus, since it contains molecules with long chains with both hydrophobic tails and hydrophilic head groups, it is believed that it can act also as a surfactant for the preparation of PLA NCs. Additionally, the presence of aldehydes (around 70% of neral and geranial) in LG essential oil can create aldol reactions [27] with the polylactic acid esters and thus lead to stable PLA/LG NCs.

The purpose of this study is to design and characterize polymeric nanospheres composed by PLA embedding LG essential oil in order obtain an efficient and sustainable nanosystem, with improved antimicrobial effects against different clinically relevant microbial species, such as: *Staphylococcus aureus*, *Pseudomonas aeruginosa*, *Escherichia coli*, and *Candida albicans* without using an additional surfactant. This system is efficient, low cost, and fast to obtain and also reduces the side effects associated with the utilization of high amounts of essential oils that may be used for therapy or prophylaxis against infectious diseases.

## 2. Materials and Methods

### 2.1. Materials and Synthesis

PLA was obtained from CorbionPurac (Amsterdam, The Netherlands) with the name Poly(dl-lactide) Purasorb grade with degradation time 9–12 months and MW 17 kg/mol. Lemongrass essential oil (100% pure) was purchased from Maitreya-Natura (Pesaro-Urbino, Italy). Acetone was purchased from Sigma Aldrich (Milano, Italy). Milli-Q water (Merck, Roma, Italy) was used for the solvent/antisolvent method for the precipitation of the NPs and NCs. To produce the PLA NPs and PLA/5-LG NCs, 100 mg of PLA were dissolved in 8 mL of acetone. To make the NCs, 5 μL of LG (5% *v*/*w* with respect to the PLA) were added into the PLA–acetone solution. Then, the solutions were dropwise dropped in 16 ml of water with a syringe. The resulting solutions were filtered using Sartorius 389F filter paper (Göttingen, Germany) and then left under nitrogen flow for 2 h to ensure complete removal of the acetone solvent.

### 2.2. Fluorescent Microscopy

The images and the emission spectrum have been obtained by a Leica TCS SP5 STED-CW (Leica Microsystems, Mannheim, Germany) inverted confocal laser scanning microscope equipped with a super-continuum laser. The images were collected using a Leica 100× HCX PL APO STED NA 1.40 Oil immersion objective (Leica Microsystems CMS, Mannheim, Germany) with an excitation at 488 nm. The emission spectrum is collected from 510 to 655 nm with an excitation at 488 nm. Detection bandwidth: 20 nm. Number of steps: 12.

### 2.3. Dynamic Light Scattering (DLS) and Z-Potential

DLS and Z-potential measurements were acquired using a Zetasizer Nanoseries from a Malvern Instruments (Worcestershire, UK) technique. The Z-average size was used in DLS and is a parameter also known as the cumulant mean, which is defined as the harmonic intensity averaged particle diameter and is hydrodynamic in nature applicable to particles in dispersion. To measure the Z-potential, the Smoluchowski Model was employed with F (Ka) value set at 1.5. The temperature was set at 25 °C and the cell type used was the DTS1060C clear disposable zeta cell. Five measurements were used with an automatic duration of a minimum of 10 and a maximum of 100 runs.

### 2.4. Raman Spectroscopy

A Horiba Jobin–Yvon µRaman operating with a He-Ne laser source (Kyoto, Japan) was used to study the molecular vibrations modes of bare LG oil, CA NPs and CA/LG NCs. The wavelength of the laser radiation was 632.8 nm and the objective used was a 50× with a slit aperture about 200 µm.

### 2.5. UV Absorption Spectroscopy

A UV-Vis-NIR spectrophotometer by Varian (Cary 6000i, Santa Clara, CA, USA) in double beam configuration was used to study the LG oil content in the PLA/5-LG NCs. First, a calibration curve was constructed by using bare LG essential oil in acetonitrile where the amount of the oil varied from 0.234 µL to 1.875 µL. The intensity values at 240 nm (which is the highest peak of LG oil absorption) for the bare LG essential oil used to obtain the absorption spectra were: 0.18, 0.48, 1.30, and 3.50. To determine the amount of LG oil in the PLA/5-LG NC solution, the measured intensity values at 240 nm were extrapolated to the calibration curve of bare LG.

### 2.6. Antimicrobial Analysis

#### 2.6.1. Microbial Strains and Growth Conditions

*Staphylococcus aureus ATCC 25923*, *Pseudomonas aeruginosa ATCC 25324*, *Escherichia coli ATCC 25922*, and *Candida albicans ATCC 10231* strains were purchased from American Type Culture Collection (ATCC, Manassas, Virginia, USA). Glycerol stocks were streaked on LB agar or Sabouraud agar (for *C. albicans*) to obtain 24 h cultures to be used for all further studies.

#### 2.6.2. Qualitative Antimicrobial Assay

For assessing the qualitative antimicrobial effects of tested compounds/nanoparticles, an adapted disc diffusion method was utilized. Microbial suspensions were obtained of each strain and adjusted to an optical density of 0.5 McFarland (1.5 × 10^8^ CFU/mL). Suspensions were utilized to inoculate the entire surface of nutritive agar in Petri dishes (or Sabouraud agar for *C. albicans*) by using a cotton swab. After inoculation, 6 mm sterile absorbent paper discs were placed on the surface of inoculated agar. Each compound was disposed on an independent sterile disc in a volume of 5 µL and Petri plates were incubated for 24 h at 37 °C to allow the microbial growth. After incubation, the diameter of the growth inhibition zone was measured (mm).

#### 2.6.3. Minimum Inhibitory Concentration (MIC Assay)

For establishing the MIC (minimum inhibitory concentration) values of the obtained compounds/nanoparticles, we utilized a microdilution method performed in nutritive broth. The sterile broth was added in sterile 96 well plates and binary dilutions of each tested compound were performed in a final volume of 150 μL (eight dilutions were investigated, namely: 1/2, 1/4, 1/8, 1/16, 1/32, 1/64, 1/128 and 1/256). After realizing the binary dilutions, 15 μL of microbial suspension adjusted to an optical density of 0.5 McFarland (1.5 × 10^8^ CFU/mL) were added in each well. The MIC values were established by naked eye analysis and spectrophotometric measurement (Abs600 nm). Each experiment was performed in triplicate and repeated on at least three separate occasions.

#### 2.6.4. Biofilm Development

Monospecific biofilm development was assessed at 24 h of treatment using a static model. Tested compounds/nanoparticles were subjected to binary dilutions in nutritive broth, utilizing an adapted microdilution method performed in sterile 96 well plates in a final volume of 150 μL. After realizing the binary dilutions, 15 μL of microbial suspension adjusted to an optical density of 0.5 McFarland (1.5 × 10^8^ CFU/mL) were added in each well. The biofilms were allowed to develop 24 h at 37 °C. After the incubation, biofilms were washed with sterile phosphate saline buffer (PBS) and fixed with cold methanol for 5 min. After methanol removal, biofilms were allowed to dry at room temperature and stained with 1.5% crystal violet for 20 min. Stained biofilms were washed with tap water, and the crystal violet embedded within the biofilm cells was released after treatment with 33% acetic acid. The absorbance of the obtained solution was measured by spectrophotometric analysis (Abs492 nm). Each experiment was performed in triplicate and repeated on at least three separate occasions.

### 2.7. In Vitro Biocompatibility Tests

The human amniotic fluid stem cells (AFSC) were used to evaluate the biocompatibility of PLA/5-LG NCs with eukaryotic cells. The AFSC were cultured in DMEM medium (Sigma-Aldrich, Darmstadt, Germany) supplemented with 10% fetal bovine serum, 1% penicillin and 1% streptomycin antibiotics (Sigma-Aldrich, City, MO, USA). The cells were used at passages 3–4 and the medium was changed twice a week to maintain optimal culture conditions. The biocompatibility was assessed using MTT assay (CellTiter 96^®^ Non-Radioactive Cell Proliferation Assay, Promega, WI, USA). This assay is based on the reduction of yellow tetrazolium salt MTT (3-(4,5 dimetiltiazolium)-2,5-diphenyltetrazolium bromide) to a dark blue formazan by the mitochondrial enzymes. Briefly, the human AFSC were grown in 96-well plates, with a seeding density of 3000 cells/well in the presence of PLA/5-LG NCs for 24–72 h. Then, 15 mL Solution I was added and incubated at 37 °C for 4 h. After that, the Solution II was added and pipetted vigorously to solubilize formazan crystals. After 1 h, the absorbance was read using spectrophotometer at 570 nm (TECAN Infinite M200, Männedorf, Switzerland). Furthermore, for confirmation of the biochemical test, a second method was used for evaluation of the biocompatibility of PLA/5-LG NCs based on fluorescent microscopy using RED CMTPX fluorescent dye (Life Technologies, Invitrogen, USA). The CMTPX is a cell tracker for long-term tracing of living cells that permit evaluation of the viability and morphology of cells in culture. The CMTPX fluorophore was added in the culture medium after 5 days in the presence of PLA/5-LG NCs at a final concentration of 5 μM, and incubated for 30 min in order to allow the dye penetration into the cells. Next, the AFSC were washed with PBS and visualized by fluorescent microscopy. The photomicrographs were taken with Olympus CKX 41 digital camera driven by CellSense Entry software (Olympus, Tokyo, Japan).

## 3. Results

As analytically described in the experimental section, the PLA NPs and PLA/5-LG NCs were produced by initially dissolving PLA in acetone and adding 5% (*v*/*w*) of LG with respect to the PLA in the second case. The NPs and NCs were formed dropwise from the acetone solutions into water. Filtration and nitrogen flow ensure purity of NPs and NCs and removal of acetone, respectively.

### 3.1. Fluorescence Microscopy

**Figure 1:**
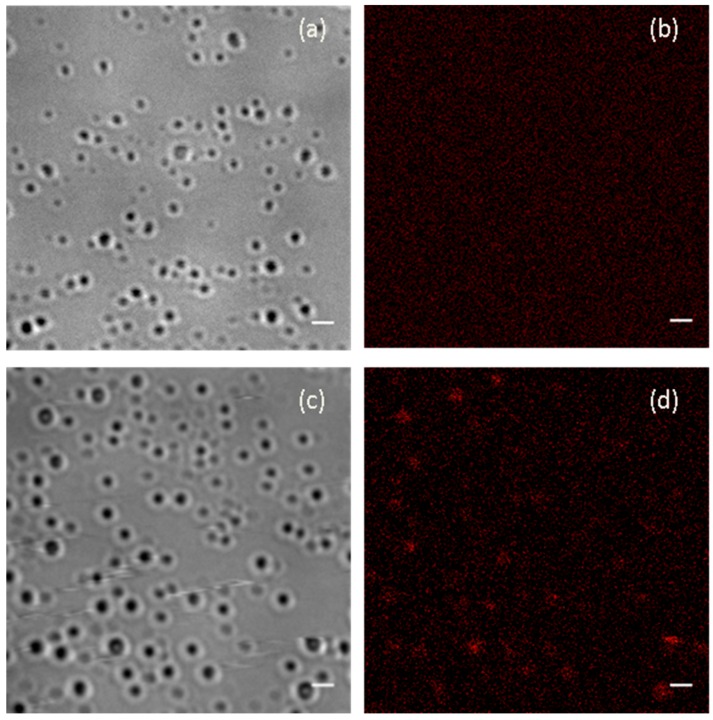
Transmission andfluorescence microscopy images of (**a**,**b**) polylactic acid nanoparticles (PLA NPs) and (**c**,**d**) polylactic acid/5-lemongrass oil nanocapsules (PLA/5-LG NCs) repsectively (scale bars, 1 µm).

Fluorescence microscopy was used to reveal the presence of lemongrass essential oil in the PLA/5-LG NCs. It was found that PLA NPs (Figure 1a) without LG were not showing any fluorescence due to the non-fluorescent nature of PLA (Figure 1b). Inversely, when LG was encapsulated into PLA NPs (Figure 1c), fluorescence was evident in the formed PLA/5-LG NCs, as shown in Figure 1d, since LG oil has fluorescent properties that retains when attached to polymers [7].

### 3.2. Dynamic Light Scattering (DLS) and Z-Potential

**Figure 2:**
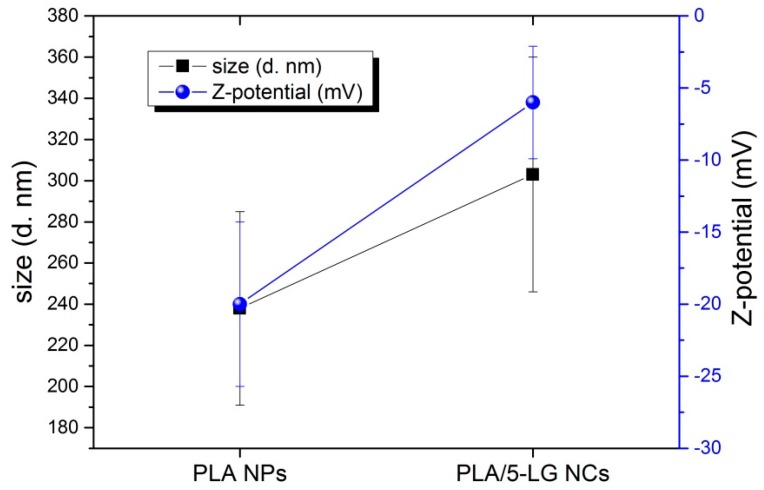
Dynamic light scattering (DLS) analysis revealing the size and Z-potential of PLA NPs and PLA/5-LG NCs.

The size of the PLA NPs and PLA/5-LG NCs was calculated using DLS. As shown in Figure 2, the average dimension of PLA NPs was about 240 ± 100 nm, while for PLA/5-LG NCs of 300 ± 110 nm with polydispersity indices (PDIs) 0.661 and 0.272, respectively. Most likely, the size of the PLA/5-LG NCs was slightly higher than that of the PLA NPs because of the encapsulation of LG essential oil that increases the average size of the NCs by about 60 nm. Z-potential of PLA NPs was around −20 mV, in agreement with other works on similar PLA NPs [28,29]. The presence of LG essential oil into PLA/5-LG NCs lowered the Z-potential to −6 mV. This decrease of the Z-potential upon grafting of the polymer with LG essential oil is in accordance with a previous work of cellulose acetate and LG essential oil [7].

### 3.3. Raman Spectroscopy

**Figure 3:**
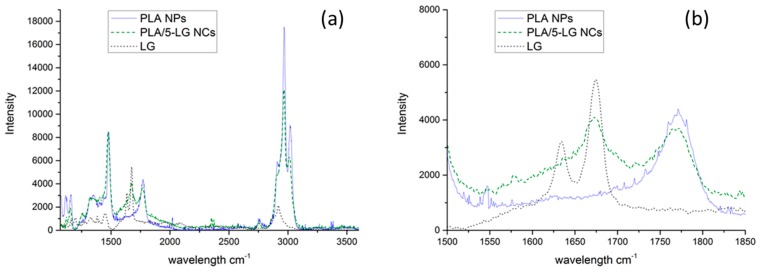
Raman spectra of PLA NPs (**blue** line), PLA/5-LG NCs (**green** dashed line) and LG oil (**black** dots) (**a**) whole spectrum and (**b**) zoomed spectrum.

Additionally, Raman spectra of PLA NPs, PLA/5-LG NCs and LG oil were acquired as shown in Figure 3a,b. LG oil has two characteristic peaks at 1625 and 1670 cm^−1^, which are associated with C=C and C=O bonds arising from the two aldehydes of neral and geranial [7,23,30]. These two peaks appeared also in the PLA/5-LG NCs (Figure 3b) with the peak at 1670 cm^−1^ being more evident. Therefore, Raman spectroscopy confirms that LG essential oil was attached to the PLA forming stable NCs.

### 3.4. UV Absorption Spectroscopy

**Figure 4:**
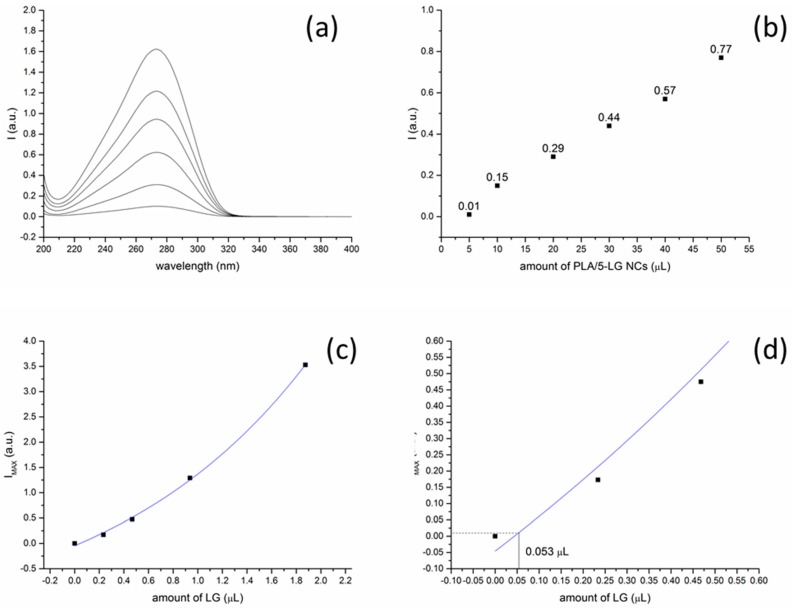
(**a**) UV absorption spectra of increasing amount of PLA/5-LG NCs solution (5, 10, 20, 30, 40 and 50 μL): the peak at 275 nm corresponds to PLA while the shoulder at 240 nm corresponds to LG absorption; (**b**) intensities of the peak at 240 nm for increasing amounts of PLA/5-LG NCs; (**c**) calibration curve of LG essential oil; and (**d**) calculation of LG oil content in 5 μL of PLA/5-LG NCs.

**Figure A1:**
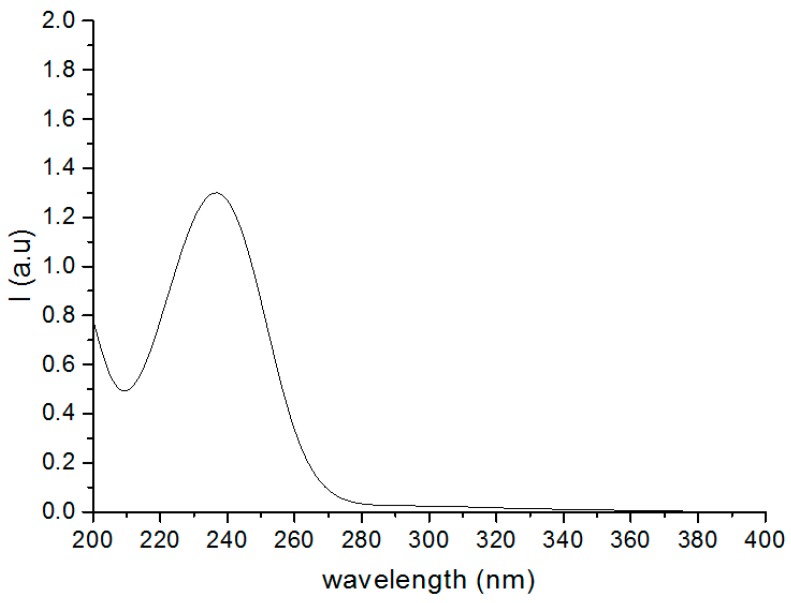
UV absorption spectra of 0.94 μL LG essential oil in 3 mL of acetonitrile.

**Figure A2:**
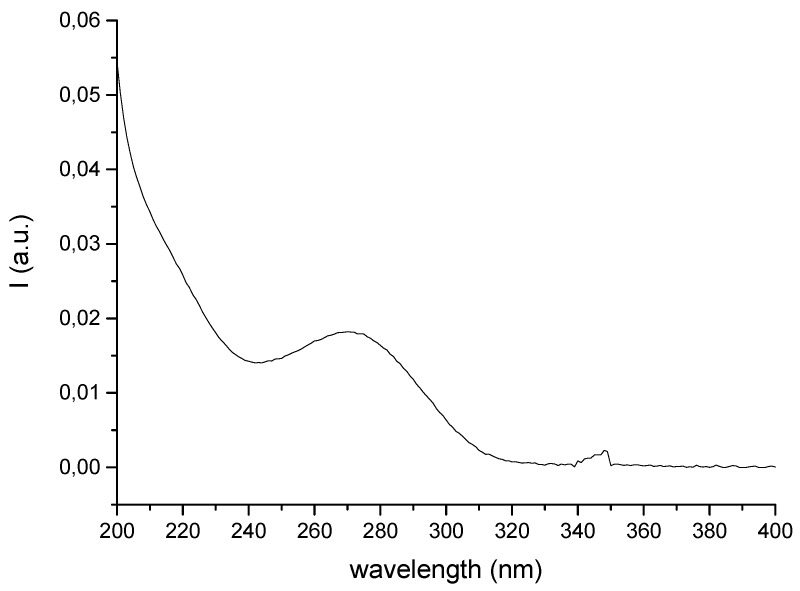
UV absorption spectra of 250 μL PLA NPs water solution in 3 mL of acetonitrile.

Before testing the aquatic PLA/5-LG NCs for their antimicrobial activity, it was necessary to calculate the amount of LG essential oil inside 5 μL of PLA/5-LG NCs aqueous solution that was subsequently used for the antimicrobial analysis. For this reason, UV spectra of increasing volume of PLA/5-LG NCs (5–50 μL) were acquired and depicted in Figure 4a. Their intensities at the top of the peak shoulder around 240 nm wavelength, originated from LG essential oil, as shown in supporting information Figure A1, were calculated and depicted in Figure 4b. The peak at 275 nm was originated from the PLA, as shown in supporting information Figure A2. It is observed from Figure 4b that the higher the amount of PLA/5-LG NCs, the higher the intensity of the peak maximum at 240 nm. Similarly, UV spectra of bare LG essential oil were acquired with increasing amount of LG, and a calibration curve of the intensity of the peaks at 240 nm approximately vs. the LG volume was constructed as shown in Figure 4c. To calculate the amount of LG in 5 μL of PLA/5-LG NC solution, the intensity of this UV spectrum at the 240 nm shoulder was extrapolated on the calibration curve, and the amount of 0.053 μL of LG was calculated (Figure 4d).

### 3.5. Antimicrobial Analysis

**Figure 5:**
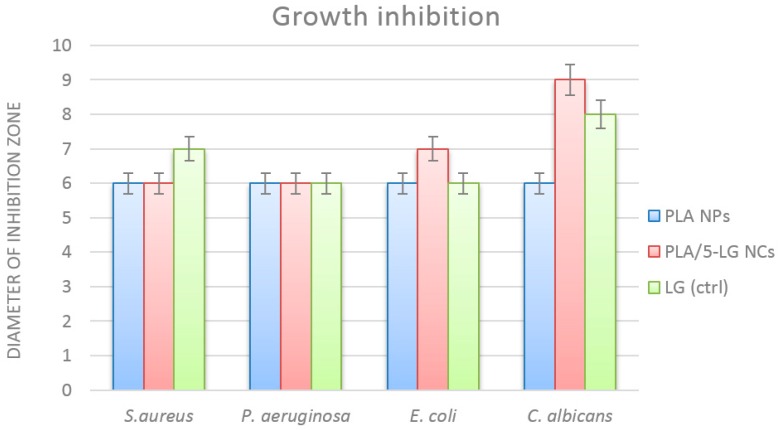
Graphic representation revealing the growth inhibition diameters of tested microbial strains grown in the presence of PLA NPs, PLA/5-LG NCs and control LG.

Antimicrobial assays proved that the obtained nanospheres were able to inhibit the growth and some virulence phenotypes (such as biofilm formation) in all tested strains, in a dose and strain-dependent manner. Qualitative test demonstrated that NCs that contain LG produce a higher growth inhibition zone in *C. albicans* and *E. coli* tested strains, as compared with plain PLA NCs (Figure 5). For the *S. aureus* and *P. aeruginosa* strains, the growth inhibition zones were minimal and remain constant for both types of tested NCs. PLA microspheres were previously utilized to deliver other natural molecules with antimicrobial effects, such as usnic acid [31]. Moreover, these nanocapsules proved their potential to be utilized in the development of anti-biofilm coatings, which can be efficiently utilized for tailoring medical devices but also in the design of antimicrobial food packaging approaches [32]. Both in *E. coli* and *C. albicans*, it can be observed that the prepared nanocapsules that contain LG essential oil are more efficient in inhibiting bacteria growth than plain LG oil, utilized at the same concentration. This may be explained by a more efficient delivery, or controlled release of the nanocapsules for the active compounds/products.

**Figure 6:**
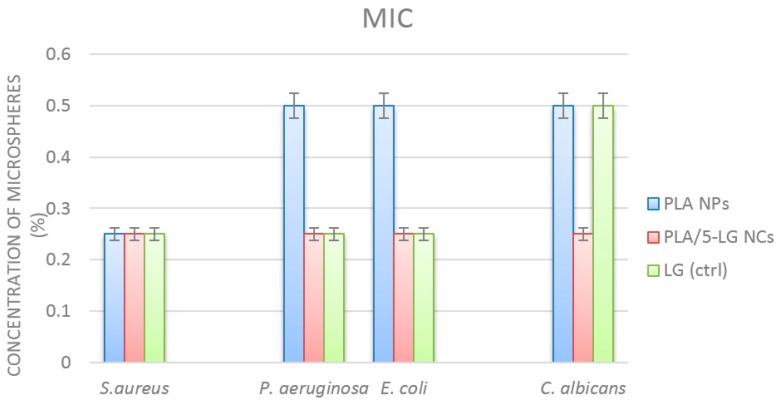
Graphic representation revealing the minimum inhibitory concentration values (MICs) of PLA NPs, PLA/5-LG NCs and control LG oil on the tested microbial strains after 24 h or incubation.

Quantitative assays revealed that PLA NCs loaded with LG essential oil have two times more pronounced antimicrobial effects, as compared with plain PLA NCs. This result is correlated with the antimicrobial effect of LG, but also with the fact that the polymeric nanocapsules are able to improve and prolong the pharmacological activity of the active compound, by stabilizing the structure, ensuring a targeted delivery and a controlled release of the active agent [33]. The minimum inhibitory concentration values were influenced also by the tested microbial strain. As revealed by Figure 6, the MIC values of PLA/5-LG NCs obtained for *P. aeruginosa*, *E. coli* and *C. albicans* was 0.25%, while the MIC value for plain PLA NPs was 0.5% (Figure 6). In *S. aureus*, the MIC concentration of both tested PLA NPs and NCs was 0.25%.

**Figure 7:**
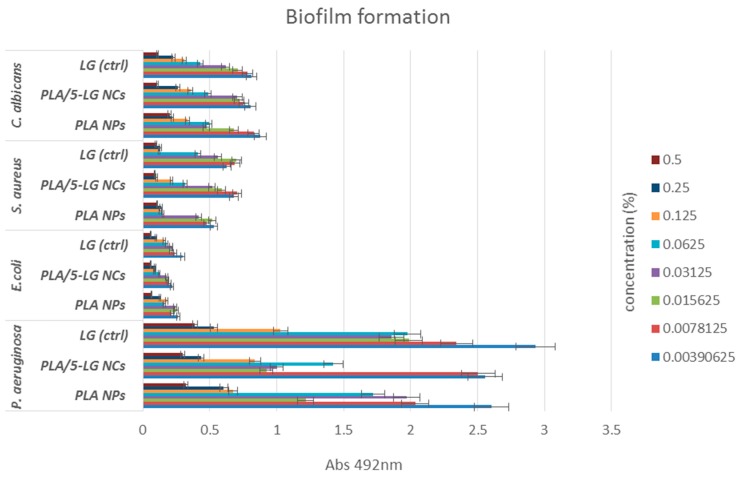
Graphic representation revealing the biofilm inhibition MICs of PLA NPs, PLA/5-LG NCs and LG oil control on the tested microbial strains.

In addition, biofilm formation assay demonstrated that the obtained NCs reduce the ability of microorganisms to develop biofilms in a dose and strain dependent manner. For most strains, biofilm inhibition was observed at concentrations ranging 0.5%–0.031% of both plain and LG loaded PLA NCs (Figure 7). The highest anti-biofilm effect was observed for *C. albicans* and *E. coli* tested strains.

The differential effects of the tested nanocapsules on various microbial strains could be due to multiple parameters. First, it is well known that, because of their great variety and versatility, microbes may behave very different in similar conditions (i.e., treatment with nanoparticles) and their distinct behavior may be explained by their bio-chemical particularities, cellular wall morphology, molecular signaling, ability to produce biofilms, etc. [34]. It is well known that, for example, the Gram character makes bacteria sensitive or resistant to different types of antimicrobial agents and conditions [35].

### 3.6. Biocompatibility Test

**Figure 8:**
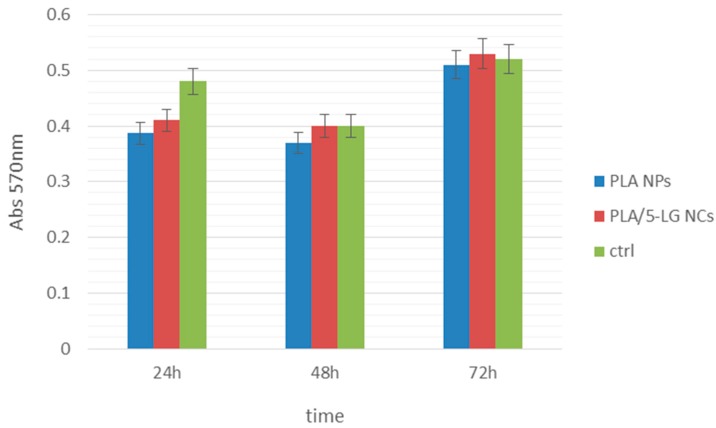
The viability of human amniotic fluid stem cells (AFSC) grown in the presence of PLA/5-LG NCs and in control conditions. The MTT assay shows that, after 72 h, the cells present a normal metabolism and growth capacity compared to control (*n* = 3, *p* < 0.05%).

The cytotoxicity effect of PLA/5-LG NCs was performed by MTT assay. This assay is based on biochemical reactions that measure the metabolic activity of living cells. The MTT assay demonstrated that the human AFSC present a normal metabolism and growth in the presence of PLA/5-LG NCs. The absorbance measurements showed a better proliferation of AFSC grown on PLA/5-LG NCs compared to those grown on PLA NPs (Figure 8).

**Figure 9:**
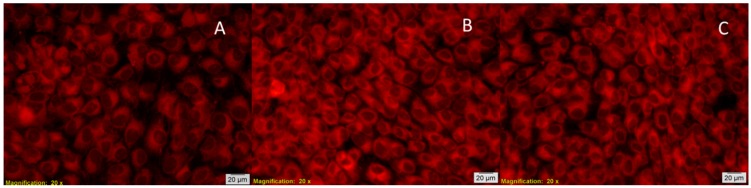
Fluorescence microscopy images of the human AFSC monolayer in the presence of (**A**) PLA NPs; (**B**) PLA/5-LG NCs and (**C**) control. The cells present a normal morphology, are adherent, and have a relative uniform distribution after five days of PLA/5-LG NC stimulation.

The fluorescence microscopy images confirm biochemical tests showing that human AFSC viability is maintained after five days in the presence of PLA/5-LG NCs. The AFSC maintains a normal morphology, and the cells are relatively uniformly distributed and adherent to culture surface (Figure 9). These results confirm the other experiments completed on macrophages that showed that lemongrass did not affect the viability of the cells [36]. The size and shape of PLA/5-LG NCs are important for the interaction with the living cells. The average dimension of PLA NPs was about 240 nm, while for PLA/5-LG NCs of 300 nm. At these sizes, the proliferation of AFSC was comparable to control samples. The in vitro assays results demonstrate a high cell viability and good growth in the present of PLA/5-LG NCs, demonstrating the biocompatibility potential of these nanocapsules and their antimicrobial properties.

## 4. Conclusions

In this study, we report on the synthesis of a type of polymeric nanocapsules made of PLA and LG essential oil. NCs were formed by using LG oil that acted as a surfactant and stabilized the PLA NCs, while exhibiting an efficient antimicrobial effect. The resulting NCs had a diameter of around 300 nm, and LG oil was proved to have been encapsulated into the PLA matrix. The potential biomedical advantages of these NCs are numerous, the most important being: their natural composition, biocompatibility, biodegradability and great dispersability in water, and also the low tendency to form clusters. Moreover, the in vitro results demonstrated that the obtained NCs have a significant antimicrobial activity and reduce biofilm formation in many clinically relevant microbial strains, such as *S. aureus*, *P. aeruginosa*, *E. coli* and *C. albicans*; therefore, they could be considered as a competitive candidate for the development of efficient antimicrobial systems active in difficult to treat infections, such as those that involve biofilm formation. Antimicrobial results, together with their physico-chemical properties and biocompatibility, recommend these nanostructures to be used for the design of efficient, personalized, and ecological drug delivery systems with great impact in antimicrobial therapy.
